# Different perspectives on women’s health, nutrition and endurance exercise

**DOI:** 10.1080/15502783.2023.2286286

**Published:** 2023-11-29

**Authors:** Nisha Charkoudian, Jason K.W. Lee, Gabrielle E.W. Giersch, Loretta DiPietro, Nina Stachenfeld

**Affiliations:** a US Army Research Institute of Environmental Medicine, Thermal & Mountain Medicine Division, Natick, MA, USA; b National University of Singapore, Heat Resilience and Performance Centre, Yong Loo Lin School of Medicine, Singapore, Singapore; c The George Washington University, Department of Exercise and Nutrition Sciences, Milken Institute School of Public Health, Washington, USA; d Yale School of Medicine, John B. Pierce Laboratory, Obstetrics Gynecology and Reproductive Sciences, New Haven, CT, USA

1.

To the editors:

We read with interest the paper by Sims et al. (2023), entitled “International society of sports nutrition position stand: nutritional concerns of the female athlete”, published in *JISSN*. We agree with, and appreciate, the authors’ emphasis regarding the relative lack of evidence regarding nutrition to optimize performance in women athletes, and the fundamental need for more research which includes sex as a biological variable (SABV). That said, we were concerned by some of the authors’ conclusions regarding recommendations for women. The purpose of this letter is to summarize these concerns, which are focused on four conclusions, italicized below:

(1) *Women are at greater risk of hyponatremia due to shifts in mechanisms of fluid regulation with estrogen and progesterone*. There is some evidence for greater incidence of hyponatremia in women in ultra-endurance type events [1–3]. However, there has been no controlled evaluation of risk; existing reports are based on correlational/regression-type analyses of field data from sporting events. More importantly, hyponatremia is rare; symptomatic, clinically relevant hyponatremia is even more uncommon. In this context, unsubstantiated implications of risk for serious illness or injury could have serious consequences as they may discourage women from participating in athletic events.

From a physiological perspective, there is no evidence that the shifts in fluid volume regulation with estradiol and/or progesterone fluctuations contribute directly to hyponatremia. Estrogens inhibit the Na-K ATPase pump, which can cause cell swelling in some circumstances in animal models [4–6]. However, this has not been demonstrated to occur in humans during or following exercise. Therefore, whether there is a biological sex-based difference in absolute risk of exercise-associated hyponatremia remains to be determined.

(2) *Women have greater deficits in thermoregulatory and cardiovascular capacity because “women have less absolute and relative fluid available to lose” during exercise in hot environments*. The only situations in which there are consistent sex differences in sweating (but not skin blood flow) is in tightly controlled laboratory conditions where very high workloads are combined with oppressive environmental conditions of heat and humidity [7]. These high workloads are not sustainable for long periods of time, especially in such environments. Therefore, assuming comparable physiological and heat acclimatization status with regular breaks and normal hydration, women and men are similar in their overall ability to thermoregulate or to maintain normal cardiovascular function and blood flow during exercise in the heat. Furthermore, in conditions of high heat and humidity, any tendency for less sweating in women is often viewed as a positive adaptation, since such sweat would be “wasted” (if it can’t evaporate to cool the body). In this sense, women may have an advantage in such conditions by having a more efficient sweating response.

3) *Variations in thirst across the menstrual cycle might be associated with cyclical effects of female reproductive hormones*. These cyclical hormone effects are minimal compared with other factors that influence thirst, such as osmolality, exercise, and body temperature. Moreover, as noted above, these hormonal shifts do not contribute to a higher risk of symptomatic hyponatremia in women compared with men.

4) *Women should tailor carbohydrate intake to hormonal status, with an emphasis on greater carbohydrate intake and availability during the luteal phase of the menstrual cycle*. There are reports suggesting that the luteal phase is associated with decreased [8], increased [9] and unchanged [10] carbohydrate utilization relative to other phases of the menstrual cycle. Thus, at the present time, there is no clear rationale to increase carbohydrate intake during any menstrual cycle phase. Furthermore, with very few exceptions, most people in the developed world (including athletes) consume adequate amounts of carbohydrates and calories. A more important public health concern is the continuing increase in carbohydrate (especially sugar) consumption and the ever-increasing prevalence of obesity observed world-wide. [11,12]

In this context, we agree that there may be a subset of people (men and women) who participate in athletic events (mostly elite/high performing athletes) and do not consume adequate carbohydrates. However, we wish to caution those women who might read this article and conclude (without evidence) that they need to consume more carbohydrates during the luteal phase of their cycle (or at all), that they should carefully calculate and monitor their energy requirements before doing so. Overall, since many/most people who regularly exercise do not perform such calculations, we feel that encouraging women to increase carbohydrate intake based on potential small fluctuations during the menstrual cycle contradicts current public health messaging.

Overall, we strongly agree with the authors that it is crucial to consider SABV and potential sex differences in physiology and nutrition research. Major funding agencies including the National Institutes of Health, the American Heart Association and the National Science Foundation now emphasize the consideration of SABV (which is separate from the study of sex differences *per se*). Furthermore, we applaud Sims and colleagues for their comprehensive synthesis of a wide range of topics relative to women and sports nutrition. The goal of the present letter is to point out areas in which readers may draw the conclusion that women are at greater risk from exercise training. Although we are certain this was not the intent of the authors, there have been instances where such assumptions about “increased risk” have resulted in the exclusion of women in sporting events [13], combat roles in the military [14], and other career or athletic endeavors.

We encourage all athletes to pay close attention to their nutritional and fluid/electrolyte requirements during training and competition. However, we do not believe that there is strong evidence in the biomedical literature to support elevated risks of hyponatremia, heat illness, low energy availability or a need for carbohydrate periodization in most women who exercise regularly and/or participate in athletic events [11,15].

## Disclaimer

Drs. Charkoudian and Giersch are employees of the U.S. Army. The opinions or assertions contained herein are the private views of the author(s) and are not to be construed as official or as reflecting the views of the U.S. Army or the Department of Defense. Citations of commercial organizations and trade names in this report do not constitute an official Department of the Army endorsement or approval of the products or services of these organizations. No conflicts of interest, financial or otherwise, are declared by the authors.
